# Influence of Bioceramic Clinker particle size, radiopacifier, and liquids on their physicochemical properties

**DOI:** 10.1590/0103-644020256326

**Published:** 2025-04-14

**Authors:** Karina Ines Medina Carita Tavares, Airton Oliveira Santos-Junior, Jáder Camilo Pinto, Fernanda Ferrari Esteves Torres, Marco Antonio Hungaro Duarte, Juliane Maria Guerreiro-Tanomaru, Mário Tanomaru-Filho

**Affiliations:** 1 Department of Restorative Dentistry, São Paulo State University (UNESP), School of Dentistry, Araraquara, São Paulo, Brazil; 2 Departament of Dentistry - Centro Universitário Presidente Antônio Carlos - UNIPAC, Barbacena, MG, Brazil, and Department of Dentistry - Centro Universitário Presidente Tancredo de Almeida Neves - UNIPTAN, São João del Rei, MG, Brazil; 3 Department of Dentistry, Endodontics and Dental Materials, School of Dentistry, University of São Paulo, São Paulo, Brazil

**Keywords:** calcium silicate, dental materials, endodontics, physical process.

## Abstract

This study evaluated the addition of calcium tungstate (CaWO_4_) as a radiopacifier and the manipulation with distilled water (DW) or liquid with additives (LA) on the physicochemical properties of Clinker (CL; Angelus) with different particle sizes (2 to 30 µm and <2 µm), compared to White MTA and MTA Repair HP. Initial and final setting times (ST) were evaluated according to ISO 6876:2012 and ASTM C266-03 standards. Radiopacity was assessed following ISO guidelines, and solubility was evaluated after immersion in distilled water or phosphate-buffered saline for 7 days. The pH was measured using a digital pH meter in 1, 3, 7, 14, 21, and 28 days. ANOVA and Tukey and unpaired *t*-tests were performed (α=0.05). CL 2 to 30 µm showed shorter initial ST and higher final ST when manipulated with LA (p<0.05). The addition of CaWO_4_ increased the CL radiopacity for both particle sizes (p<0.05) but did not influence ST, solubility, and pH (p>0.05). All materials presented alkaline pH (p>0.05), especially in 24 hours and 7 days. Regarding the solubility, all groups exhibited mass gain, while White MTA in distilled water demonstrated mass loss (p<0.05). CL 2 to 30 µm + CaWO_4_ showed greater mass gain and higher radiopacity when manipulated with LA than DW (p<0.05). CL <2 µm manipulated with LA provides a shorter final ST. Both particle sizes positively influence the alkalization capacity of CL, especially in the initial periods. The addition of calcium tungstate and manipulation using LA showed adequate physicochemical properties for clinical application.

## Resumo

O objetivo deste estudo foi avaliar a adição de tungstato de cálcio (CaWO_4_) como radiopacificador e a manipulação com água destilada (AD) ou líquido com aditivos (LA) nas propriedades físico-químicas do Clínquer (CL; Angelus) com diferentes tamanhos de partículas (2 a 30 µm e <2 µm), em comparação com o MTA Branco e o MTA Repair HP. O tempo de presa (TP) inicial e final foi avaliado de acordo com as normas ISO 6876:2012 e ASTM C266-03. A radiopacidade foi avaliada conforme as normas ISO, e a solubilidade foi avaliada após imersão em água destilada ou solução salina com fosfato por 7 dias. O pH foi mensurado com pHmetro digital em 1, 3, 7, 14, 21 e 28 dias. Os testes ANOVA e Tukey e o teste t não pareado foram realizados (α=0,05). CL 2 a 30 µm apresentou menor TP inicial e maior TP final quando manipulado com LA (p<0,05). A adição de CaWO₄ aumentou a radiopacidade do CL para ambas as partículas (p<0,05), porém não influenciou o TP, a solubilidade e o pH (p>0,05). Todos os materiais apresentaram pH alcalino (p>0,05), principalmente nas 24 horas e 7 dias. Quanto à solubilidade, todos os grupos apresentaram ganho de massa, enquanto MTA Branco em água destilada mostrou perda de massa(p<0,05). CL 2 a 30 µm + CaWO₄ apresentaram maior ganho de massa e maior radiopacidade quando manipulados com LA do que com AD (p<0,05). CL <2 µm manipulado com LA proporcionou menor TP final. Ambos os tamanhos de partículas influenciam positivamente na capacidade de alcalinização do CL, principalmente nos períodos iniciais. A adição de tungstato de cálcio e a manipulação com LA apresentaram propriedades físico-químicas adequadas para aplicação clínica.

**Figure:**
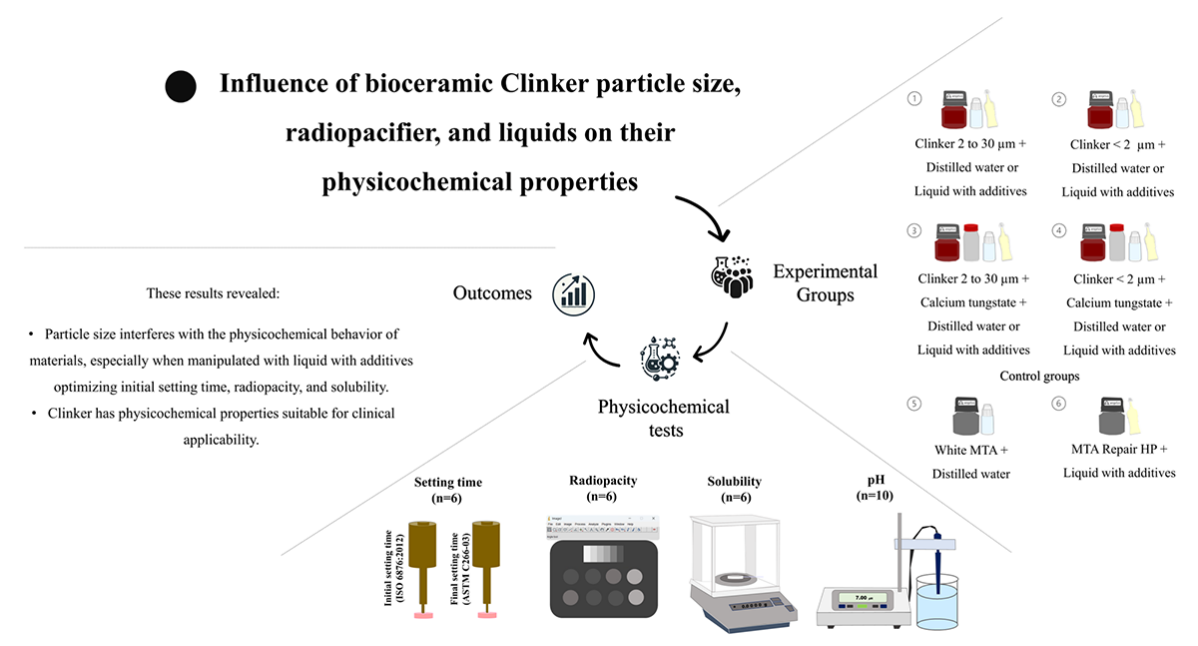

## Introduction

Materials based on calcium silicates, such as Mineral Trioxide Aggregate (MTA) from Angelus (Angelus® Indústria de Produtos Odontológicas S/A, Londrina, PR, Brazil) are made with raw materials manufactured from pure compounds using calcium carbonate, aluminum oxide and silicon dioxide. Subsequently, an electric kiln is used to sinter the powder called Clinker with different particle sizes (2 to 30 µm or < 2 µm), providing a lower risk of contamination ^1^. Next, the Clinker is subjected to a grinding process, responsible for forming fine-grain particles of the material, which are subsequently associated with the radiopacifier agent. The association of pure tricalcium silicate with calcium tungstate as a radiopacifier provides excellent biological, antimicrobial ^2^, physical ^2^ ^,^ ^3^, and mechanical properties ^4^.

The particle size can influence the performance of endodontic materials (^5^ ^,^ ^6^ ^,^ ^7^ ^,^ ^8^). In the presence of larger particles, the hydration of the material is incomplete, compromising the setting time, alkalization capacity, compressive strength, and interaction of the material with the dentin ^5^. On the other hand, smaller particles provide a shorter setting time, improve the flow performance, and enhance the compressive strength of the cement ^7^. Furthermore, when the material or radiopacifier powder has smaller particles (1.5 µm or 0.8 µm), a greater surface area is established, favoring the physicochemical, mechanical, and biological properties of calcium silicate-based cement ^5^ ^,^ ^7^ ^,^ ^8^.

Material handling involves mixing the powder with liquid. The establishment of an adequate proportion allows for obtaining a homogeneous mixture, favoring the physicochemical properties of hydraulic materials ^9^. A high amount of water impairs the dimensional stability of White MTA ^10^. Furthermore, the incorporation of organic agents in distilled water aims to improve the handling and insertion of the material ^11^. However, greater solubility, porosity, and gaps in the material/dentin interface have been reported when using liquid with additives ^11^ ^,^ ^12^.

Particle size, radiopacifiers, and manipulation of repair materials and their interactions have a direct impact on the long-term success of treatments. In this context, Clinker-based bioceramics deserve to be studied. Although previous studies have examined the isolated advantages and disadvantages of these factors ^2^ ^,^ ^3^ ^,^ ^4^ ^,^ ^5^ ^,^ ^6^ ^,^ ^7^ ^,^ ^8^ ^,^ ^13^ ^,^ ^14^, it is essential to assess their synergistic impact on the physicochemical properties of the material, particularly when it is associated with Clinker. Therefore, this study aimed to evaluate the addition of calcium tungstate as a radiopacifier and the manipulation with distilled water (DW) or liquid with additives (LA) on the physicochemical properties of Clinker (Angelus) with different particle sizes (2 to 30 µm and < 2 µm), compared to White MTA and MTA Repair HP (Angelus). The null hypothesis proposed was that no significant differences would be found in the physicochemical properties among the evaluated materials.

## Materials and methods

The materials with their respective manufacturers, compositions, and proportions are described in Table 1. Pilot studies were performed to adequately determine the powder/liquid proportion for each experimental group.

**Table 1:** Experimental groups, material with their manufacturers, composition, and proportion

Groups	Material and Manufacturer	Composition	Proportion
CL 2 to 30 µm + DW or LA	Clinker 2 to 30 µm Angelus, Londrina, PR, Brazil Lot: 61186	Powder: tricalcium silicate, dicalcium silicate, tricalcium aluminate and calcium oxide Liquid: distilled water or liquid with additives (water and polyvinylpyrrolidone)	1g powder : 300 μL distilled water 1g powder : 300 μL liquid with additives
CL ˂ 2 µm + DW or LA	Clinker ˂ 2 µm Angelus, Londrina, PR, Brazil Lot: 60578	Powder: tricalcium silicate, dicalcium silicate, tricalcium aluminate and calcium oxide Liquid: distilled water or liquid with additives (water and polyvinylpyrrolidone)	1g powder : 530 μL distilled water 1g powder : 530 μL liquid with additives
CL 2 to 30 µm + CaWO_4_ + DW or LA	Clinker 2 to 30 µm + 30%CaWO_4_ Angelus, Londrina, PR, Brazil Lot: 61186	Powder: tricalcium silicate, dicalcium silicate, tricalcium aluminate, calcium oxide and calcium tungstate Liquid: distilled water or liquid with additives (water and polyvinylpyrrolidone)	1g powder : 230 μL distilled water 1g powder : 230 μL liquid with additives
CL ˂ 2 µm + CaWO_4_ + DW or LA	Clinker ˂ 2 µm + 30%CaWO_4_ Angelus, Londrina, PR, Brazil Lot: 60578	Powder: tricalcium silicate, dicalcium silicate, tricalcium aluminate, calcium oxide and calcium tungstate Liquid: distilled water or liquid with additives (water and polyvinylpyrrolidone)	1g powder : 390 μL distilled water 1g powder : 390 μL liquid with additives
Mta	White MTA Angelus, Londrina, PR, Brazil	Powder: tricalcium silicate, dicalcium silicate, tricalcium aluminate, calcium oxide and calcium tungstate Liquid: distilled water	1g powder: 330 μL distilled water
MTAHP	MTA Repair HP Angelus, Londrina, PR, Brazil	Powder: tricalcium silicate, dicalcium silicate, tricalcium aluminate, calcium oxide and calcium tungstate Liquid with additives: water and polyvinylpyrrolidone	1g powder : 300 μL liquid with additives

CL: Clinker, CaWO_4_:Calcium tungstate, DW: Distilled water, LA: Liquid with additives, MTAHP: MTA Repair HP

### Physicochemical tests

### Sample Size Calculation

The G*Power 3.1.7 program for Windows (Heinrich-Heine- Universität Dusseldorf, Dusseldorf, Germany) was used for sample calculation. One-way ANOVA was used with an alpha-type error of 0.05, and a beta power of 0.95 for all variables. The specific effect size for each variable was calculated: 2.83 for setting time, 2.11 for radiopacity, 0.96 for pH, and 1.68 for solubility ^15^. Six specimens per group were indicated as the ideal size required.

### Setting Time

Setting time was evaluated according to the International Organization for Standardization (ISO) 6876:2012 ^16^ and American Society for Testing and Materials (ASTM) C266-03 ^17^. After manipulation, the materials were inserted into plaster models (Type IV; Durone IV Salmon; Dentsply, Petropolis, RJ, Brazil) with a 10 mm internal diameter and 1 mm height (n=6). A Gilmore needle of 100 ± 0.5 g and 2 ± 0.1 mm diameter was used to determine the initial setting time, as established by the ISO 6876 standard. Subsequently, a Gilmore needle of 456 ± 0.5 g and 1. 0 ± 0.1 mm diameter was used to determine the final setting time, as established by ASTM C266-03. The initial and final setting times were considered in minutes, from the manipulation of the materials until the moment when the respective needles could not produce marks on the surface of the cement.

### Radiopacity

After manipulation, the materials were inserted into circular plastic molds with a 10 mm internal diameter and a 1 mm height (n=6) and stored in an oven for 24 hours. After this period, the disc of each material and an aluminum scale with thickness ranging from 2 mm to 16 mm, in 2 by 2 step wedges, were placed on an occlusal film (Insight - Kodak Comp., Rochester, NY). A radiographic image (GE 1000 X-ray machine - General Electric, Milwaukee, WI) was performed using the following parameters: 60 kV, 7 mA, 0.32 pulses per second, and a focus-film distance of 33 cm. The films were processed, digitized, and evaluated using the Image J software (National Institutes of Health, Bethesda, USA).

### Solubility

Circular plastic molds were made with 1.5 mm in height and 7.75 mm in internal diameter ^18^ and filled with each material (n=6), inserting a nylon thread included in the mixture of each cement. The set was stored in an oven at 37 °C for 24 hours. The specimens were removed from the molds and weighed on a precision balance (Adventurer AR2140; Ohaus Corporation, Parsippany, NJ, USA) to determine the initial mass. Subsequently, the samples for each experimental group were suspended using nylon threads inside plastic containers with lids containing 7.5 mL of distilled and deionized water or phosphate-buffered saline. The containers remained in an oven at 37 °C for 7 days. After this period, the specimens were removed from the solutions, rinsed, and placed in a desiccator with silica. The samples were weighed every 24 hours until the final mass stabilized (0.001g). The percentage of solubility was determined using the following formula: [Percentage of solubility = Initial mass - final mass /initial mass * 100] ^2^ ^,^ ^13^.

### pH

Polyethylene tubes with open extremities (10 mm high and 1.6 mm diameter) were filled with each material (n=10). Each tube was immersed in 10 mL of distilled and deionized water in a flask with a lid and kept in an oven at 37 °C. After each experimental period, the tubes were placed in a new flask with 10 mL of distilled and deionized water. The experimental periods were 1, 7, 14, 21 and 28 days. The pH of the solutions was measured using a previously calibrated digital pH meter (Mettler Toledo, Columbus, Ohio, USA). Flasks containing only distilled and deionized water were used as a control group.

**Figure 1:**
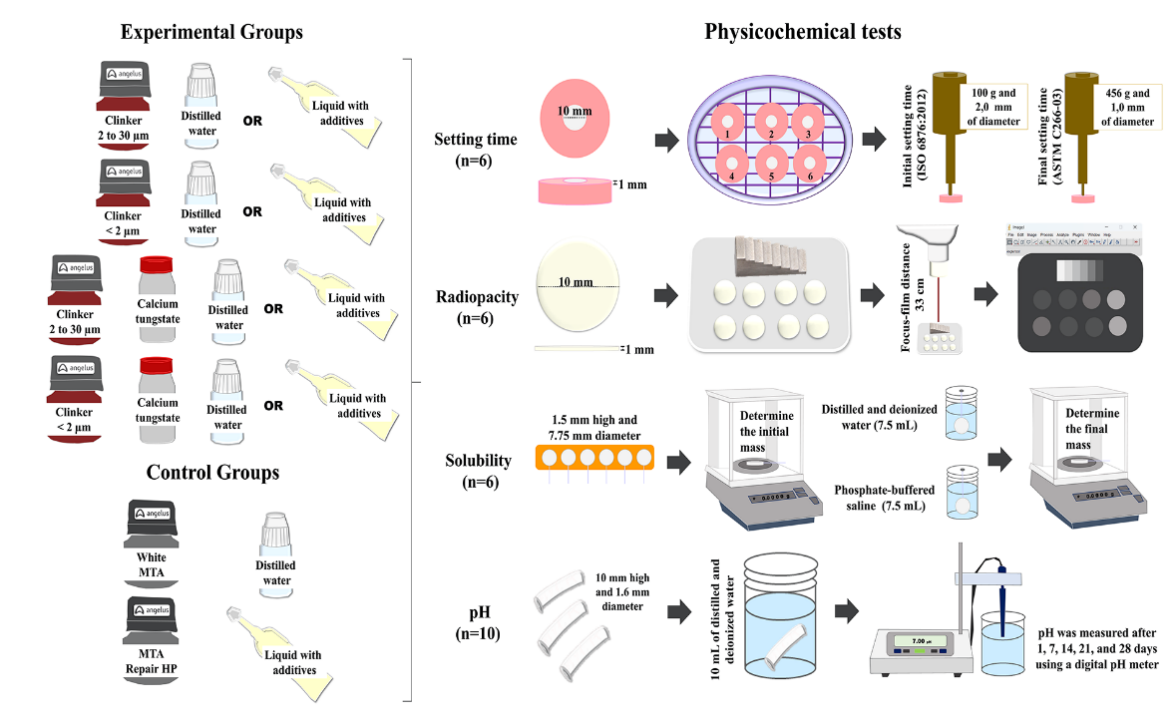
Conceptual framework of the methodology employed to assess the physicochemical properties (setting time, radiopacity, solubility, and pH) of different calcium silicate-based repair materials

Figure 1 presents the conceptual framework of the study design used to assess the physicochemical properties of different calcium silicate-based repair materials.

### Statistical analysis

All data were submitted to the Shapiro-Wilk test and showed normal distribution. The One-way ANOVA and Tukey tests were used for comparisons between groups. To compare the different pH periods, repeated measures ANOVA and Tukey were used. The unpaired *t-*test was used for comparisons between distilled water or liquid with additives and comparisons between immersion solutions. The level of significance was 5% for all analyses.

## Results

Clinker 2 to 30 µm showed shorter initial setting time and longer final setting time when manipulated with LA (p<0.05) (Table 2). Clinker groups manipulated with DW showed shorter initial setting time compared to LA (p<0.05) (Table 2). Clinker < 2 µm exhibited a shorter final setting time when manipulated with LA (p<0.05), while the addition of calcium tungstate significantly prolonged the initial setting time compared to MTA Repair HP (p<0.05). White MTA showed a longer initial and final setting time than clinker groups manipulated with DW, and a longer initial setting time compared to MTA Repair HP (p<0.05).

**Table 2:** Mean and ±standard deviation of the initial and final setting time, radiopacity and solubility of different calcium silicate repair materials

	CL 2 to 30 µm	CL ˂ 2 µm	CL 2 to 30 µm + 30 % CaWO_4_	CL ˂ 2 µm + 30 % CaWO_4_	MTA	MTA HP
Initial setting time (min.)						
DW	15.0 ± 0.4^bB^	11.6 ± 2.5^bB^	18.3 ± 2.5^bB^	12.5 ± 2.7^bB^	41.5 ± 11.5^aA^	-------
LA	19.5 ± 2.5^dA^	30.5 ± 3.3^abA^	24.5 0.54^cdA^	33.3 ± 4.63^aA^	------	26.1 ± 4.2^bcB^
Final setting time (min.)						
DW	166.0 ± 18.5^bB^	121.1 ± 0.4^cA^	200.3 ± 13.4^bA^	107.8 ± 0.4^cB^	245.4 ± 49.3^aA^	------
LA	211.3 ± 6.8^aA^	115.6 ± 13.0^bA^	181.3 ± 6.7^aB^	126.6 ± 0.5^bA^	------	190.5 ± 52.5^aA^
Radiopacity (mmAl)						
DW	1.85 ± 0.02^bA^	1.83 ± 0.04^bA^	3.80 ± 0.39^aB^	3.57 ± 0.22^aA^	4.01 ± 0.60^aA^	-------
LA	1.88 ± 0.04^bA^	1.80 ± 0.01^bA^	4.42 ± 0.18^aA^	3.99 ± 0.49^aA^	-------	3.97 ± 0.59^aA^
Solubility (%)						
Immersion solution - Distilled water						
DW	-3.50 ± 1.92^bA*^	-4.10 ± 1.55^bA*^	-2.36 ± 1.88^bA*^	-3.62 ± 1.14^bA*^	3.21 ± 1.97^aA*^	-------
LA	-6.45 ± 2.75^aA^	-4.52 ± 2.39^aA^	-4.11 ± 1.41^aB*^	-1.98 ± 1.65^aA*^	-------	-4.54 ± 1.68^aB^
Immersion solution - Phosphate buffer saline						
DW	-10.4 ± 2.06^abA*^	-7.53 ± 2.75^abA*^	-6.77 ± 1.19^bA*^	-11.47 ± 4.64^aA*^	-9.94 ± 1.73^abA*^	--------
LA	-8.17 ± 2.91^aA^	-6.91 ± 3.17^aA^	-9.83 ± 1.86^aB*^	-7.06 ± 2.08^aA*^	-------	-7.12 ± 2.97^aA^

CL: Clinker, CaWO_4_: calcium tungstate, MTAHP: MTA Repair HP, Handling liquid (DW: Distilled water or LA: Liquid with additives)

Negative values in the solubility test indicate mass gain and positive values indicate mass loss

Different superscript lowercase letters in the same line indicate a significant difference between groups (p<0.05). Different superscript uppercase capital in the same column indicates a significant difference between the different liquids for manipulation (p<0.05). * Indicates a significant difference between immersion solutions for each handling liquid (p<0.05).

The addition of calcium tungstate influenced the Clinker radiopacity for both particles (p<0.05) and showed similar radiopacity to White MTA and MTA Repair HP (p>0.05). However, the radiopacifier did not influence the setting time, solubility, and pH (p>0.05) (Table 2).

All materials showed mass gain (p>0.05), while White MTA showed mass loss when immersed in distilled water (p<0.05). Clinker 2 to 30 µm + calcium tungstate showed greater mass gain and higher radiopacity when manipulated with LA than DW (p<0.05). Distilled water provided less mass gain compared to phosphate-buffered saline as an immersion solution (p<0.05) (Table 2).

All materials presented alkaline pH (p>0.05). After 24h and 21 days, Clinker < 2 µm with and without calcium tungstate and manipulated with DW or LA showed higher alkalinity of the medium than White MTA (p<0.05). Clinker < 2 µm promoted higher pH than Clinker 2 to 30 µm in some periods and groups (p<0.05) (Table 3).

**Table 3:** Mean and ±standard deviation of the pH values obtained of the calcium silicate repair materials in different experimental periods

	Groups	DW	DW + 30 % CaWO_4_	LA	LA + 30 % CaWO_4_	MTAHP	MTA	Control
24 hours	CL 2 to 30 µm	10.07 ± 0.35^abA^	10.16 ± 0.18^abA*^	10.30 ± 0.12^aA^	10.04 ± 0.32^abA*^	9.78 ± 0.81^bA^	-------	7.17 ± 0.18^cA^
	CL ˂ 2 µm	10.44 ± 0.24^aA^	10.41 ± 0.25^aA*^	10.18 ± 0.52^abA^	10.56 ± 0.22^aA*^	-------	10.05 ± 0.2^bA^	7.16 ± 0.13^cA^
7 days	CL 2 to 30 µm	8.85 ± 0.44^aB^	9.68 ± 0.35^aB^	9.74 ± 0.41^aB*^	9.83 ± 0.50^aA^	8.86 ± 0.83^bB*^	-------	6.68 ± 0.29^cB*^
	CL ˂ 2 µm	9.38 ± 0.69^aB^	9.57 ± 0.44^aB^	10.17 ± 0.06^aA*^	9.52 ± 0.47^aB^	-------	9.89 ± 0.58^aA*^	7.16 ± 0.08^bA*^
14 days	CL 2 to 30 µm	8.29 ± 0.24^aB*^	8.60 ± 0.28^aC*^	8.46 ± 0.27^aC*^	8.58 ± 0.41^aB*^	8.50 ± 0.53^aB*^	-------	6.77 ± 0.11^bB*^
	CL ˂ 2 µm	8.87 ± 0.52^bBC*^	9.05 ± 0.46^bC*^	9.78 ± 0.09^aA*^	9.40 ± 0.38^abB*^	-------	8.98 ± 0.26^bB*^	7.02 ± 0.18^cA*^
21 days	CL 2 to 30 µm	7.91 ± 0.29^bC*^	7.92 ± 0.13^bD*^	7.94 ± 0.13^bD*^	8.27 ± 0.39^abB*^	8.34 ± 0.49^aB^	-------	6.76 ± 0.07^cB*^
	CL ˂ 2 µm	8.76 ±0.40^bcBC*^	8.94 ± 0.28^bD*^	9.04 ± 0.25^abB*^	9.31 ± 0.26^aB*^	-------	8.41 ± 0.28^cC^	6.23 ± 0.13^dB*^
28 days	CL 2 to 30 µm	8.40 ±0.13^abBC*^	8.09 ± 0.03^bD^	8.12 ± 0.17^bD*^	8.62 ± 0.36^aB^	8.34 ±0.46^abB*^	-------	6.65 ± 0.27^cB^
	CL ˂ 2 µm	8.24 ± 0.30^bC*^	8.31 ± 0.41^bD^	8.62 ± 0.32^abB*^	8.36 ± 0.58^bC^	-------	9.05 ± 0.34^aB*^	6.43 ± 0.46^cB^

CL: Clinker, CaWO_4_:calcium tungstate, MTAHP: MTA Repair HP, Handling liquid (DW: Distilled water or LA: Liquid with additives), Control: distilled and deionized water. Different superscript lowercase letters in the same line indicate a significant difference between groups (p<0.05). Different superscript uppercase letters in the same column indicates a significant difference between the different experimental periods (p<0.05). * Indicates statistical difference in the particle size of Clinker (p<0.05).

## Discussion

This study evaluated the physicochemical properties of Clinker Angelus in comparison to White MTA and MTA Repair HP, focusing on the influence of particle size, the addition of a radiopacifier agent, and the use of different manipulation liquids. Understanding the combined effects of these factors is crucial for optimizing material performance in clinical practice. A previous systematic review ^8^ highlighted the limited evidence available on calcium silicate-based cement with smaller particles, emphasizing the need for further research. Based on the differences observed in physicochemical properties in this study, the null hypothesis was rejected.

The LA provided a shorter initial setting time for Clinker 2 to 30 µm, which can be related to changes in the hydration process of the material by the presence of the plasticizer in the water ^2^, accelerating the setting ^19^. However, the final setting time for Clinker 2 to 30 µm was longer when compared to Clinker < 2 µm. This finding may be related to particle size, since larger particles have a smaller surface area, providing prolonged setting time ^6^. Another factor related to setting time is the powder/liquid ratio used to handle repair materials ^10^. In the present study, all materials manipulated with DW, except White MTA, showed shorter initial setting times when compared to LA. Conversely, a previous study reported a longer setting time when materials were handled with DW ^2^. These differences may be related to material handling ^10^, and particle size with a direct impact on the setting reaction of calcium silicate-based materials ^5^ ^,^ ^7^. Achieving a balance in the setting time is important since a very short setting time affects the working time ^20^, while a longer setting time may increase the solubilization and dissolution of endodontic materials ^11^.

Adequate radiopacity is crucial for ensuring that the material can be clearly distinguished from adjacent anatomical structures during radiographic evaluations ^21^. Our results demonstrated that all experimental groups, except for Clinker without the addition of calcium tungstate, exhibited radiopacity values in compliance with ISO 6876:2012 standards, which require a minimum radiopacity of 3 mm of aluminum ^16^. Comparable radiopacity values were observed across the Clinker, White MTA, and MTA Repair HP groups, corroborating findings from previous studies ^2^ ^,^ ^13^. Different radiopacifier concentrations modify hydraulic materials' properties ^3^. The addition of 30% calcium tungstate to tricalcium silicate provides satisfactory physicochemical and biological properties ^2^ ^,^ ^3^. Therefore, in this study, 30% calcium tungstate was added to the Clinker, providing adequate radiopacity for both particles without changes in the other physicochemical properties evaluated. The current results indicate that the LA positively influences the radiopacity of Clinker 2 to 30 µm. We can suggest that the polyvinylpyrrolidone used with the plasticizer of the MTA Repair HP liquid may have accelerated the hydration of the material and reduced the dissociation of the calcium tungstate. Furthermore, organic agents optimize the physical properties of calcium silicate-based materials ^14^ ^,^ ^20^.

In the present study, all materials showed alkalinization capacity when compared to the control group, especially during the periods of 24 hours and 7 days. The alkalinity of Clinker may contribute to its biocompatibility and antimicrobial activity ^22^. Furthermore, a pH of 7.8 to 8.0 is considered ideal for obtaining cell migration and consequently favors apical repair ^23^. Moreover, in this study, it was pointed out that Clinker < 2 µm promoted a higher pH compared to Clinker 2 to 30 µm. Disagreeing with our results, a previous study reported that the addition of smaller particles decreases the release of hydroxyl ions ^7^. This result may be related to the sintering process of calcium silicate powder and the powder/liquid ratio used in the present study.

High solubility of materials can compromise seal integrity, promoting bacterial recontamination ^10^. In our study, all materials exhibited mass gain, while White MTA exhibited mass loss. The balance between calcium ion release and solubility must occur to ensure material integrity ^10^. Calcium silicate-based cements have hydrophilic properties, promoting continuous water absorption ^10^ ^,^ ^13^ and mass increase. However, there are no standardized parameters for mass increase of repair materials. Although the liquid with additives improves physicochemical properties, it also increases solubility. ^11^. Higher solubility was reported for MTA Repair HP (8.18%) when compared to MTA Angelus (4.91%) ^11^. Our results showed that manipulation using a liquid with additives favored the solubility of Clinker 2 to 30 µm associated with calcium tungstate, in accordance with a previous study ^13^.

The ISO methodology for solubility by mass loss assessment has limitations for hydraulic cement ^24^ ^,^ ^25^. The water absorption capacity during the setting process and the drying of the samples during the evaluation can significantly affect the evaluation ^24^. Simulated body fluids promote results closer to clinical reality ^24^. In our study, the materials demonstrated greater mass gain in phosphate-buffered saline compared to distilled water. This result is related to the water sorption potential of the material and the interaction between calcium ions from the Bioceramic Clinker and phosphate from the phosphate-buffered saline solution ^25^. This interaction facilitates the formation of a surface layer of hydroxyapatite, which contributes to the increase in the mass of the material.

White MTA and MTA Repair HP are commonly used as reference materials due to their physicochemical properties and clinical relevance ^2^ ^,^ ^3^ ^,^ ^4^ ^,^ ^5^ ^,^ ^9^ ^,^ ^10^ ^,^ ^11^ ^,^ ^12^ ^,^ ^13^ ^,^ ^19^ ^,^ ^20^. Despite having similar particle sizes and powder compositions, the liquid influences their properties ^2^ ^,^ ^14^ ^,^ ^19^ ^,^ ^20^. In the present study, White MTA demonstrated longer initial and final setting times than Clinker manipulated with DW. This difference can be attributed to variations in particle size and powder/liquid ratio. Smaller particles can accelerate the hydration process, reducing the setting time ^6^. Furthermore, a larger volume of water prolongs the setting time ^2^ ^,^ ^10^. On the other hand, MTA Repair HP demonstrated a shorter initial setting time compared to White MTA and Clinker < 2 µm combined with calcium tungstate and manipulated with DW. The plasticizer incorporated into the MTA Repair HP liquid reduces the setting time ^19^, although it potentially increases the solubility and porosity of the material ^11^ ^,^ ^19^. Our results showed that White MTA presented lower alkalinization capacity than Clinker < 2 µm after 24 h and 21 days. This result is related to the hydration process and release of calcium ions that occur more rapidly in smaller particles ^8^. However, White MTA maintained an alkaline pH throughout all evaluation periods ^11^ ^,^ ^13^. These observations highlight the influence of particle size, powder composition, and liquid handling on the physicochemical properties of these materials.

In general, these results revealed that particle size interferes with the physicochemical behavior of materials, especially when manipulated with LA optimizing initial setting time, radiopacity, and solubility. Furthermore, our results suggest that Clinker has physicochemical properties suitable for clinical applicability. One limitation of this study is that all evaluations were performed *in vitro*, which may not fully replicate the complex variables present in a clinical environment. Therefore, it is essential to conduct further analyses focusing on the biological properties of these materials. Future *in-vivo* studies are recommended to provide a more comprehensive understanding of the behavior of Clinker Angelus under clinical conditions.

## Conclusion

Clinker < 2 µm manipulated with LA provides a shorter final setting time. Both particle sizes influence the alkalization capacity of Clinker, especially in the initial periods. The addition of calcium tungstate and manipulation with LA showed adequate physicochemical properties for Clinker.
